# Standard clinical approaches and emerging modalities for glioblastoma imaging

**DOI:** 10.1093/noajnl/vdac080

**Published:** 2022-05-26

**Authors:** Joshua D Bernstock, Sam E Gary, Neil Klinger, Pablo A Valdes, Walid Ibn Essayed, Hannah E Olsen, Gustavo Chagoya, Galal Elsayed, Daisuke Yamashita, Patrick Schuss, Florian A Gessler, Pier Paolo Peruzzi, Asim K Bag, Gregory K Friedman

**Affiliations:** Department of Neurosurgery, Brigham and Women’s Hospital, Harvard Medical School, Boston, Massachusetts, USA; Medical Scientist Training Program, University of Alabama at Birmingham, Birmingham, Alabama, USA; Department of Neurosurgery, Brigham and Women’s Hospital, Harvard Medical School, Boston, Massachusetts, USA; Department of Neurosurgery, Brigham and Women’s Hospital, Harvard Medical School, Boston, Massachusetts, USA; Department of Neurosurgery, Brigham and Women’s Hospital, Harvard Medical School, Boston, Massachusetts, USA; Department of Neurosurgery, Brigham and Women’s Hospital, Harvard Medical School, Boston, Massachusetts, USA; Department of Neurosurgery, University of Alabama at Birmingham, Birmingham, Alabama, USA; Department of Neurosurgery, University of Alabama at Birmingham, Birmingham, Alabama, USA; Department of Neurosurgery, University of Alabama at Birmingham, Birmingham, Alabama, USA; Department of Neurosurgery, Unfallkrankenhaus Berlin, Berlin, Germany; Department of Neurosurgery, University of Rostock, Germany; Department of Neurosurgery, Brigham and Women’s Hospital, Harvard Medical School, Boston, Massachusetts, USA; Department of Diagnostic Imaging, St. Jude Children’s Research Hospital, Memphis, Tennessee, USA; Department of Neurosurgery, University of Alabama at Birmingham, Birmingham, Alabama, USA; Division of Pediatric Hematology and Oncology, Department of Pediatrics, University of Alabama at Birmingham, Birmingham, Alabama, USA; Comprehensive Cancer Center, University of Alabama at Birmingham, Birmingham, Alabama, USA

**Keywords:** glioblastoma (GBM), mass spectrometry, MRI, PET, radiographic progression, tumor progression

## Abstract

Glioblastoma (GBM) is the most common primary adult intracranial malignancy and carries a dismal prognosis despite an aggressive multimodal treatment regimen that consists of surgical resection, radiation, and adjuvant chemotherapy. Radiographic evaluation, largely informed by magnetic resonance imaging (MRI), is a critical component of initial diagnosis, surgical planning, and post-treatment monitoring. However, conventional MRI does not provide information regarding tumor microvasculature, necrosis, or neoangiogenesis. In addition, traditional MRI imaging can be further confounded by treatment-related effects such as pseudoprogression, radiation necrosis, and/or pseudoresponse(s) that preclude clinicians from making fully informed decisions when structuring a therapeutic approach. A myriad of novel imaging modalities have been developed to address these deficits. Herein, we provide a clinically oriented review of standard techniques for imaging GBM and highlight emerging technologies utilized in disease characterization and therapeutic development.

Gliomas are primary brain tumors that arise from glial cells or neuroglial progenitors and account for approximately 80% of adult central nervous system malignancies.^1^ Glioblastoma (GBM, WHO grade IV) is the most frequently occurring subtype, with an incidence of 3.2 per 100,000 people and a median survival of 14.6 months when receiving the current standard of care.^1,2^ Survival estimates may be longer for select patients with favorable molecular profiles or those who are able to comply with tumor-treating fields (TTFields) therapy.^3,4^ GBM’s uniformly poor prognosis has remained largely unchanged despite decades of research into tumor biology and hundreds of clinical trials. Treatment shortcomings can be attributed to intratumoral heterogeneity, inability to completely resect infiltrating tumor edges, rapid development of treatment-resistant pathways, and pharmacokinetic limitations due to poor blood-brain-barrier (BBB) permeability.

The current standard of care for GBM is treatment with maximal safe surgical resection, post-operative radiation, and chemotherapy, followed by adjuvant temozolomide chemotherapy.^2,5,6^ Tumors invariably recur after treatment, with no standardized protocol for managing recurrence. Efforts to better understand the molecular pathogenesis of GBM and to extend lifespan have translated into only three FDA approved treatments—temozolomide, bevacizumab, and TTFields—all of which have minimal impact on overall survival.^7–9^ Recent research strategies have shifted to targeting GBM with immunotherapy-based approaches, though a number of phase III trial failures have left the field fairly stagnant.^5,6,8–21^

Radiographic evaluation plays a crucial role in managing patients with GBM, both during initial diagnosis and following treatment. Magnetic resonance imaging (MRI) is the modality of choice for diagnosis and assessment of treatment response due to its wide availability, and superior soft tissue visualization over computed tomography (CT). The interpretation of imaging studies after treatment of GBM is challenging because treatment-related changes frequently mimic tumor progression (termed “pseudoprogression”). Similarly, some biologically directed therapies can mask the presence of persistent lesions (termed “pseudoresponse”).^22^

It is important to understand what imaging can offer in diagnosis and management of GBM so that the right imaging techniques can be employed. Herein, we review the prospects and limitations of various imaging techniques with data in the evaluation of GBM available in most academic medical centers.

## Imaging for Diagnosis of GBM

### Magnetic Resonance Imaging (MRI)

#### Conventional MRI.—

**Figure 1.**
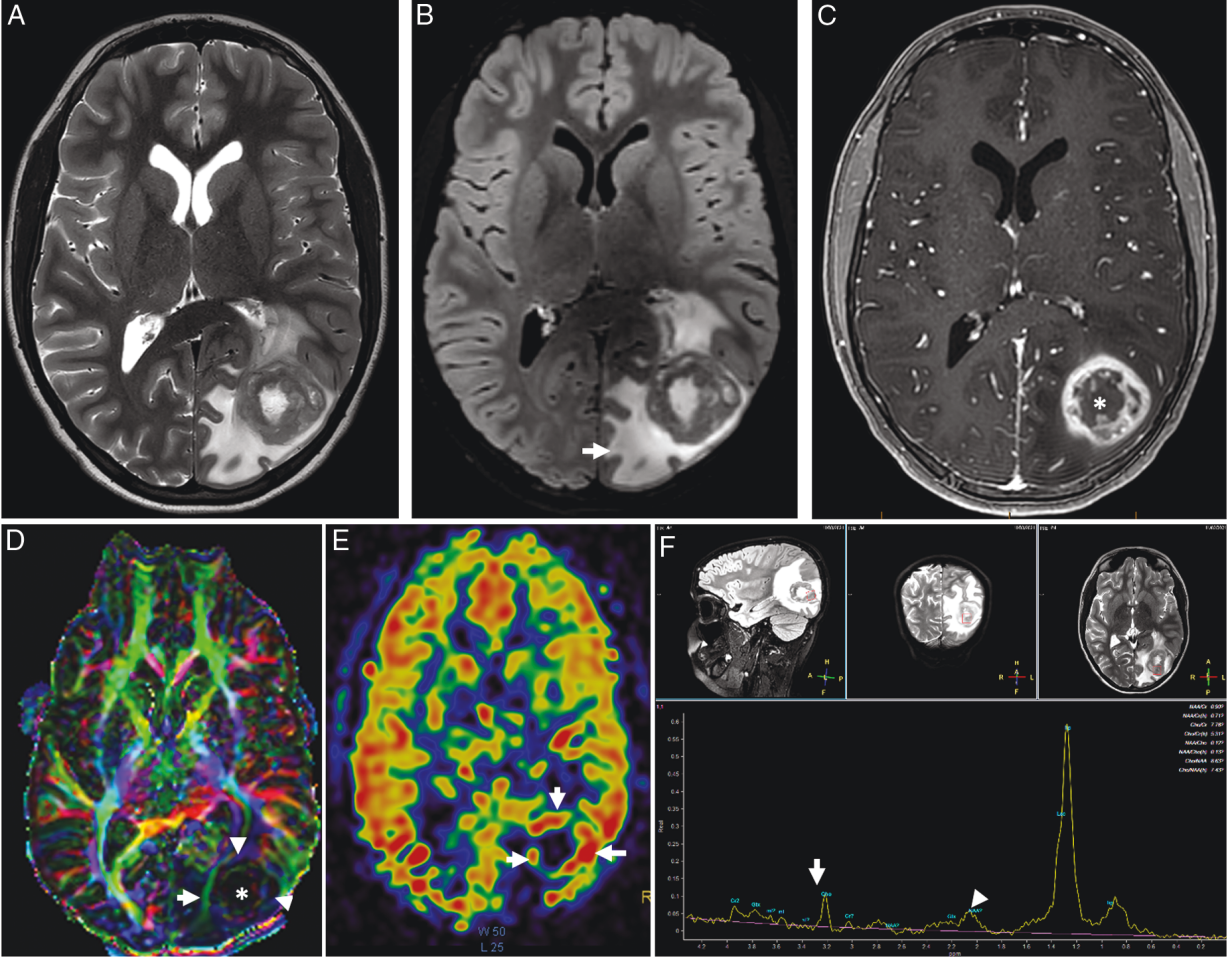
Typical imaging appearance of glioblastoma in a 12 year old male subject. Axial T2-weighted (A) and fluid attenuated inversion recovery (FLAIR) (B) images demonstrate a heterogeneously T2 hyperintense tumor centered at the left occipital lobe with extensive peritumoral edema (arrow). Axial post contrast T1 weighted imaging demonstrating classic heterogenous, rim enhancing lesion (C). The color coded fractional anisotropy image (D) through the tumor demonstrates distrupted brain architecture at the necrotic tumor core (*). The left inferior longitudinal fasciculus is thinned out and displaced due to the mass effect of the tumor. The arrowheads at the tumor periphery demonstrate low intensity compared to the contralateral hemisphere suggesting, lower FA values. Cerebral blood flow (CBF) from 3D PCASL arterial spin labelling perfusion imaging (E) demonstrates high blood flow at the peripheral enhancing component of the tumor (arrows). The single voxel MR spectroscopy (F) demonstrates severely truncated choline (arrow) and N-Acetyl Aspartate (NAA) peaks (arrowhead) as well as a dominant lipid/lactate peak. This is a typical imaging feature when a spectroscopy voxel includes both tumor and necrotic tissue.

**Figure 2.**
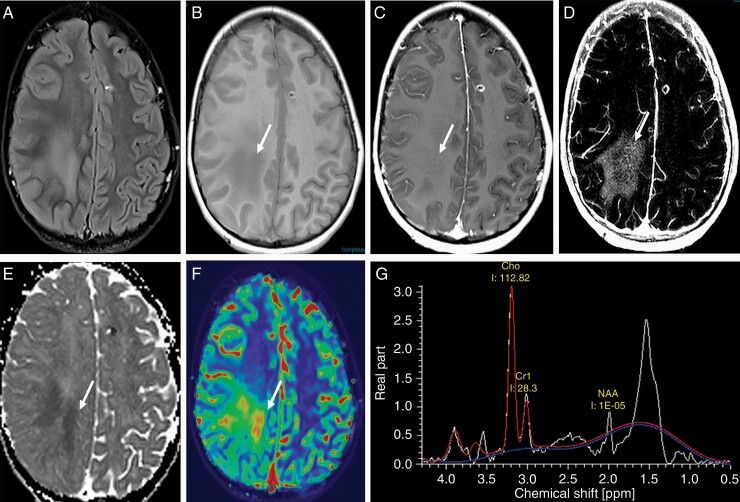
Importance of advanced imaging in the evaluation of GBM. Axial T2 FLAIR images (A) through the level of the centrum semiovale demonstrate patchy areas of FLAIR hyperintensity in the deep cerebral white matter that are hypointense (arrow) on the pre-contrast T1-weighted sequence (B) with imperceptible enhancement (arrow) on the post contrast T1-weighted sequence (C). The enhancement is vivid (arrow) on the delta T1 map (D) that also demonstrates low (arrow) ADC value (E) suggesting hypercellularity; high (arrow) cerebral blood volume (CBV) (F) and a tall choline peak compared to NAA on MR spectroscopic imaging (G).

T2-weighted, fluid attenuated inversion recovery (FLAIR) and pre- and post-gadolinium T1-weighted MRI are routinely used for diagnosis, pre-operative surgical planning, and evaluation of treatment response in patients with GBM.^23^ GBM characteristically presents on imaging as a heterogeneous enhancing mass with a central necrotic core and peritumoral edema.^2,6,9,20,21^ Associated features such as tumor volume, peritumoral edema, necrosis, degree of enhancement, and presence of cysts are additional parameters that can be used to predict outcomes and survival in patients with GBM^24^ (Figure 1A, 1B, 1C). Subtraction of the pre-contrast T1 weighted sequence from the identical post-contrast T1 weighted sequence, also known as the delta T1 map, can be very helpful for assessment of enhancing component of the tumor (Figure 2D). Intratumoral susceptibility signal (ITSS) on susceptibility weighted imaging (SWI) can readily identify intratumoral hemorrhage, neoangiogenesis, and calcification.^25^ ITSS score correlates well with the cerebral blood volume (CBV)^26,27^ and high ITSS score is more common in larger GBMs and GBMs arising from or in close proximity to the subventricular zone.^28^

Diffusion weighted imaging (DWI),^2^ an MRI sequence that assesses cellular architecture by measuring the Brownian motion of water in tissue, has shown promise in predicting tumor grade, peritumoral infiltration, and treatment-related effects in GBM. The apparent diffusion coefficient (ADC) represents the average magnitude of diffusion at the voxel or region of interest level and has been shown to negatively correlate with the cellularity of brain tumors^29–31^ (Figure 2E). ADC values of around 1000 × 10 mm^2^/s and less using routine *B* values (*b* = 0, 1000 s/mm^2^) can differentiate high grade gliomas from low grade gliomas with high sensitivity and specificity.^32,33^ ADC values may also be used to predict the genetic architecture of a GBM. IDH mutated tumors have significantly higher ADC values compared to the IDH wild-type tumors.^34^ Additionally, some studies have used ADC to predict prognosis, whereas low tumor ADC is associated with shorter survival times.^35,36^

Evaluation of GBM with qualitative DWI has become standard, however, the significance of DWI images should be interpreted carefully. The accuracy of DWI can be significantly affected by hemorrhage, which is frequently present in GBMs. In addition, high-grade gliomas have been shown to upregulate aquaporin channels, notably AQP4 and AQP1, limiting the ability of ADC to truly capture restricted diffusion in a hypercellular environment.^37–39^ Moreover, ADC is a scalar quantity that measures isotropic diffusion of water and therefore lacks information regarding anisotropy, precluding ADC from accurately differentiating areas of tumor infiltration from peritumoral edema and healthy nervous tissue.

**Figure 3.**
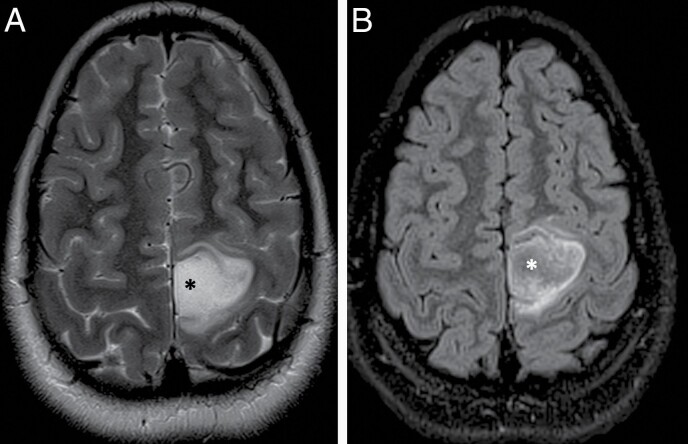
T2-FLAIR mismatch sign in an 11 year old female patient with a diagnosis of IDH-mutant, 1p/19q non-codeleted and p53-muted anaplastic astrocytoma in the left parietal lobe. (A) Axial T2-weighted image demonstrating an ill-defined tumor in the left paracentral lobule region with almost homogenous hyperintense T2 signal at the center of the tumor (*) that is mostly hypointense on the corresponding FLAIR image (B).

Recently, many conventional imaging features have been identified that may serve as unique features in certain biomarkers of GBM. T2-FLAIR mismatch sign (Figure 3), homogenously T2 hyperintense tumor with central hypointensity on FLAIR, can reliably predict IDH mutated 1p/19q non-co-deleted GBMs.^40,41^ On the other hand, high ITSS score on SWI is associated with IDH wildtype and MGMT unmethylated GBMs.^42^

As previously alluded to, diagnosis of GBM using conventional imaging alone is not always straightforward. Many other brain pathologies (e.g., tumefactive multiple sclerosis) can mimic GBM on conventional imaging sequences and can be difficult to differentiate from GBM. As such, conventional imaging in combination with other advanced imaging techniques have better diagnostic performance and have been developed for this reason.

#### Advanced MRI Techniques.—

Advanced MRI techniques, when available, should be routinely used for evaluation of GBM, both at diagnosis and during follow-up. Diffusion imaging, perfusion imaging, and MR spectroscopy (MRS) provide more detailed information about the tumor physiology and metabolism that is essential to make an informed decision about tumor management (Figure 2). Additionally, diffusion tensor imaging and functional imaging are very helpful for surgical planning.

##### Diffusion MRI

###### Diffusion Tensor Imaging.

Diffusion tensor imaging (DTI) is another powerful diffusion imaging tool that can detect more intricate tissue microarchitecture by tensor modeling of diffusion data to provide anisotropy and diffusivity measurements. DTI is often obtained pre-operatively to reconstruct white matter tracts in peritumoral regions, enabling surgeons to assess tumor infiltration and viability of peritumoral nervous tissue.^43^ Multiple studies have demonstrated increased progression-free survival and retention of functionality using DTI tractography to guide tumor resections.^35,44,45^ The concurrent use of DTI with other methods such as functional-MRI (fMRI) and direct electrical stimulation to assess brain function when constructing a surgical plan permits more robust analysis of white matter integrity.

Its benefits notwithstanding, DTI tractography faces several limitations due to the complex pathology and microinfiltrative nature of GBM. The tendency of gliomas to invade adjacent white matter disrupts the diffusion of water in affected tracts,^46–51^ resulting in a decreased fractional anisotropy, which may be modeled as an abrupt termination of the tract^52,53^ and lead surgeons to incorrectly conclude that healthy nervous tissue is unsalvageable. Furthermore, DTI tractography is unable to model the geometric complexity of the microscopic nervous architecture such as crossing fibers, which may result in incorrect or incomplete reconstructions. Like all imaging obtained preoperatively, the use of DTI for intraoperative guidance is complicated by intraoperative brain shift. This may result in significant discrepancies between neuronavigation and the intraoperative anatomy.^54–56^ Finally, significant disagreement can be encountered between DTI and the intraoperative gold standard for identifying functional white matter tracts, direct electrical stimulation. Novel diffusion MRI techniques are being developed to overcome DTI’s limitations and have shown promise in characterizing tumoral vs. healthy white matter and correlating diffusion metrics to tumor histology in both adult and pediatric brain tumor specimens.^57–59^ Nevertheless, DTI currently serves as a valuable adjunct imaging method, with its limitations understood in light of other more accurate intraoperative adjuncts such as direct electrical stimulation.^54,55^

##### Perfusion MRI

###### Dynamic Susceptibility Contrast (DSC) Perfusion Imaging.

DSC-MR perfusion relies on dynamic loss of susceptibility-induced signal during the passage of a bolus of highly paramagnetic gadolinium based contrast through a capillary bed. A dynamic echo-planar imaging sequence with a very high temporal resolution (<5 s for whole brain coverage) is used to scan the brain before, during and after injection of contrast. The dynamic signal intensity of each voxel is plotted against time, thereby generating a signal intensity vs. time curve. Using mono-compartmental modelling, this curve is then utilized to compute multiple perfusion parameters with or without incorporating the ∆R2* function. As disruption of the blood brain barrier is almost universal in GBMs, these parameters need to be corrected to account for contrast leakage. Of the many parameters, cerebral blood volume (CBV) is most widely used, which correlates with vascular endothelial proliferation, vascular density, and neoangiogenesis and has been shown to reliably correlate with histopathologic grading and survival outcomes in glioma patients^60^ (Figure 2F).

DSC-MRI is an attractive option given its short acquisition time and simple postprocessing but is nevertheless subject to several limitations. Most notably, the fundamental basis of this technique is based on a single compartment vascular model (i.e., contrast stays within vessels), which is rarely the case in GBMs. Indeed, disruption of the BBB is routinely seen in GBMs, so rCBV measurements should be performed with proper leakage correction techniques.^61,62^

###### Dynamic Contrast Enhancement (DCE) Perfusion Imaging

DCE-MR perfusion is a technique that estimates cerebral perfusion parameters by evaluating T1 shortening induced by a contrast bolus passing through tissue. The most common parameter used in tumor imaging is the volume transfer constant (*K*^trans^), which is a measure of microvascular permeability. *K*^trans^ has diagnostic power in determining glioma grade, with increased *K*^trans^ values reflecting a higher degree of BBB damage and vascular compromise.^63^ DCE-MRI is not subject to susceptibility artifacts and has therefore been used to differentiate pseudoprogression from recurrent GBM with 85% sensitivity and 79% specificity.^64^ The major drawback of this method, as compared to the DSC referenced above is the sparse literature confirming imaging via histopathologic validation and a notable lack of FDA-approved/validated post-processing software.

###### Arterial Spin Labeling (ASL) Perfusion Imaging

ASL is an MR perfusion technique that does not require intravenous administration of an exogenous contrast agent and instead uses arterial water as an endogenous tracer to measure cerebral blood flow (CBF). ASL has proven utility as a noninvasive method of determining glioma grade and distinguishing between treatment effects and tumor recurrence in postoperative gliomas.^65–67^ CBF has also been shown to correlate with IDH and ATRX mutation status, which are of prognostic relevance.^68^ ASL has a low signal to noise ratio and, unlike DSC perfusion, does not require leakage correction. However, like other dynamic perfusion techniques, ASL is limited by poor spatial resolution (Figure 1E).

##### MR Spectroscopy (MRS)

MRS is a non-invasive imaging modality used to interrogate the tissue metabolic environment.^69,70^ The main principle underlying MRS is that the distribution of electrons within an atom will cause small variations in the magnetic field experienced by different molecules, shifting the detected resonance frequency. Common neurobiological substrates include lactate, alanine, N-acetylaspartate (NAA), creatinine (Cr), choline (Cho), 2-hydroxyglutarate (2-HG), and glutamine/glutamate, and brain tumors demonstrate markedly different spectra than healthy brain tissue (Figure 1F, 2G). High grade gliomas classically show decreased levels of NAA and creatinine and increased levels of choline, lipids, and lactate.^71^ In addition, 2-HG MRS can be used to determine the IDH-mutant status of low-grade gliomas and GBM.^72,73^ MRS also can identify distinct GSCs subclones with unique metabolic phenotypes using unsupervised agglomerative hierarchical clustering, a machine-learning approach for uncovering novel biomarkers that may be used for personalized therapies.^74^ Similarly, using MRS-guided analysis of GBM metabolomes to predict the likelihood of early response of chemotherapeutic drugs such as temozolomide is an exciting area of future study.^75^ Some authors also report that MRS can be helpful in distinguish oligodendroglioma from astrocytoma.^76^ While MRS has been used to assess *in vivo* brain tumors, its applications have been limited to supplemental characterization, and as such this technique has not been widely adopted in clinical practice; this is due in part due to poor spatial resolution and large overlaps in metabolic profiles (e.g., between tumor and radiation necrosis).

##### Functional MRI

Functional magnetic resonance imaging (fMRI) indirectly measures neuronal activity via the blood oxygen level dependent (BOLD) signal, a measure of the ratio of oxyhemoglobin to deoxyhemoglobin.^77,78^ Gray matter is relatively well-vascularized, enabling fMRI to assess function and preservation of underlying neurons in cortical areas. Due to fMRI’s dependence on activity-induced changes in adjacent vasculature and not the neurons themselves, direct assessments of structural information, e.g., neuronal integrity and microstructural architecture, are impossible using BOLD fMRI alone.

Its limitations notwithstanding, fMRI is a valuable tool for surgical planning and in particular, when deciding on awake vs. asleep procedures. It can help direct cortical mapping to decrease the case length and time under anesthesia, and aid in cortical preservation when awake surgeries are impossible or fail. The combination of fMRI and DTI increases the accuracy of the preoperative planning across modalities.^46,48,79–83^ As stated above, DTI tractography may incorrectly reconstruct tracts in the presence of glioma-induced changes in diffusion of peritumoral white matter tracts, as well as shifting of tissue after resection.^48,84^ In addition, DTI’s modelling of the diffusion tensor currently limits identification of crossing fibers, leading to potential tractography errors in highly complex subcortical areas. The addition of fMRI as a seeding tool for DTI tractography and as a confirmatory tool to assess functionally sensitive areas serves to minimize DTI’s shortcomings.

The utility of fMRI for preoperative planning of brain tumor resection has recently expanded to include assessment of resting-state (non-task-based) activity of neural networks.^85,86^ Resting-state fMRI (rs-fMRI) records fluctuations in brain activity as a result of spontaneous metabolic changes and has been shown to correlate with underlying anatomic networks in the somatomotor cortices^87^ and language areas.^88^ Rs-fMRI has several advantages over fMRI in the setting of GBM. First, rs-fMRI does not depend on motor and cognitive activity, which may be affected in patients with GBM. Second, rs-fMRI can be used in combination with anesthesia to assess neural function in patients who may not comply with motion restrictions inside of a scanner (e.g., pediatric patients). Finally, rs-fMRI simultaneously records activity from multiple areas of the brain that fire spontaneously, eliminating the necessity to perform multiple tasks to assess multiple areas of the brain.

Despite the aforementioned, direct electrical stimulation remains the gold standard in identifying functional gray and white matter tissue, highlighting significant limitations with fMRI.^54,55^ Guisanni et al^89^ found that fMRI had specificities and sensitivities ranging from 0% to 97% and 59% to 100%, respectively, when compared to direct electrical stimulation for language mapping. Further, fMRI does not always inform the surgeon of areas of the brain that can be safely resected due to the brain’s limited ability to compensate without them (e.g., fMRI positive areas that when stimulated intraoperatively with direct electrical stimulation do not induce a transient deficit). Therefore, pre- and intraoperative decision making based solely on fMRI can lead to patient under selection as well as increased likelihood for limited tumor resection leaving significant amounts of tumor infiltrated tissue behind.^55^

### PET Imaging

Positron emission tomography is an imaging technique that involves intravenous injection of a positron-emitting radioisotope followed by detection of radioactivity to analyze patterns of accumulation and distribution throughout the body. In the oncology setting, PET provides valuable physiologic information not obtainable by conventional MR and CT imaging methods, and PET has utility in tumor grading, spatial reconstruction, surgical planning, post-treatment monitoring, and prognostication.

^18^F-2-fluoro-2-deoxy-D-glucose (^18^F-FDG) is the most widely studied and commonly used radiotracer.^18^F-FDG is a glucose analog and its uptake into cells is dependent upon both transport across the BBB and metabolism within tumor cells, which provides information on differentiation and specificity. In line with other malignant tumors, GBM is highly^18^F-FDG avid due to its increased glycolytic metabolism and cellular proliferation. However, normal brain tissue has a relatively high rate of physiologic glucose metabolism, leading to regional variation that may confound accurate radiographic interpretation. Contrast-enhanced MR imaging is often performed in tandem with FDG-PET and the resulting images are fused to allow for tumor co-localization^90^. FDG-PET shows high sensitivity in differentiating high grade gliomas from other types of brain tumors and has also been shown to correlate with survival and time to tumor progression^91–94^. However, due in part to poor distinguishability of tumor from normal brain, FDG-PET alone is rarely used in routine clinical practice for evaluation of intrinsic brain tumors.

**Figure 4.**
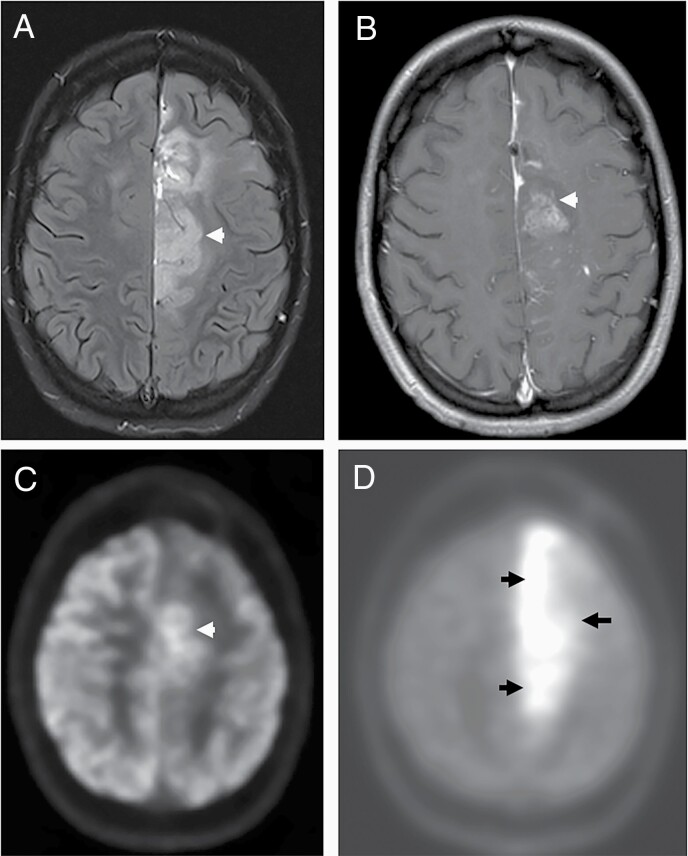
Metabolic imaging of glioblastoma at recurrence. (A) Axial T2 FLAIR images through the level of the centrum semiovale demonstrate a large, ill-defined, heterogenous tumor involving the body of the left cingulate gyrus that demonstrates patchy enhancement on the post contrast T1-weighted sequence. (B)^18^FDG PET (C) demonstrates hypermetabolism involving only the anterior aspect of the tumor, whereas the ^11^C-Methionine PET and (D) depicts the entirety of the tumor more conspicuously.

Large neutral amino acid (LNAA) PET tracers are an alternative class of radiotracers commonly used for clinical studies in glioblastoma and include 11C-MET, 11C-AMT, 18F-FET, and 18F-FDOPA.^95^ These compounds demonstrate high uptake in glioma cells and low uptake in inflammatory and normal cerebral tissue. They are therefore more useful in delineating tumoral boundaries, which are classically underestimated by conventional MRI and obscure with FDG PET due to high background metabolic activity^96,97^ (Figure 4).

Dynamic FET PET is a powerful imaging tool that relies upon sequential scans to provide information on glioma grading^92^ and prognosis.^98^ Pharmacokinetic analysis yields physiologic data regarding uptake mechanisms and tumoral heterogeneity that allows for further differentiation and superior tumor grading prediction as compared to static scanning.^92^ This method can detect high-grade transformation in lesions otherwise presenting as suspected LGGs, and can be used to predict methylation status ^99,100^. As such, dynamic FET scanning has immense potential and may ultimately be employed to assess IDH1/2 mutational and 1p/19q co-deletion status ^101^.

## Imaging During Treatment of GBM

### Intraoperative MRI (iMRI)

Gross total resection is the mainstay of GBM surgical management, with the extent of resection (EOR) correlating to symptomatic improvement and to significant improvement in both progression free survival and overall survival. EOR is largely dependent upon tumor proximity to, and infiltration of, eloquent brain regions with a high risk of neurologic comorbidities conferred by a necessarily aggressive surgical approach. Innovative technological developments have better enabled surgeons to balance maximizing EOR and preserving function. In particular, the advent of intraoperative MRI (iMRI) has led to enhanced safety and noninvasive visualization of tumor location and adjacent structures with immediate radiographic feedback prior to closure. iMRI is associated with a greater extent of gross total resection and a quantitative increase in EOR as compared to conventional neuronavigation methods ^16–18,20,21,102–104^. While iMRI was associated with increased EOR and gross total resection in a multicenter retrospective study of 640 adult patients with newly diagnosed glioblastoma, it was not a predictor of overall survival ^105^. Therefore, further research is needed to determine the full benefit of iMRI. Despite its successes in increasing EOR and gross total resection, iMRI faces several limitations, as reviewed by Kubben et al.^106^ and has not been widely implemented outside of a limited number of high-volume centers. Namely, iMRI is extremely cost prohibitive, with an individual machine in the range of several million dollars with additional costs incurred from ancillary equipment and specialized training required for operating room personnel. Further, iMRI provides only a one time (per imaging scan) immediate feedback of the surgical field of view, but similar to pre-operative MRI, once surgery resumes, any intraoperative brain shift that occurs can lead to significant discordance between the iMRI images and the intraoperative reality due to registration errors. To combat this, hybrid iMRI and 3D ultrasound techniques are being evaluated to compensate for brain shift in intraoperative navigation ^107^. Finally, iMRI has been shown to be time consuming with a significant increase in the surgical time, limiting the number of scans obtained during the same surgical setting to one or two scans in most centers. It does not, however, appear to increase perioperative risk with respect to infection or complications arising from prolonged time under anesthesia ^108,109^.

### Intraoperative Ultrasound (iUS)

Intraoperative ultrasound (iUS) is a cheap, rapid, and repeatable imaging technique used since the 1980s to maximize EOR and functional protection postoperatively. In addition, iUS is appealing because it can be implemented several times to correct for brain shift throughout resection, but the literature is unclear on whether it is efficacious for detecting and grading glioma and tumor remnants during/after surgery as compared to other imaging methods. Standard gray-scale (B mode) iUS has been the most widely implemented iUS method and has been shown to reveal tumor areas as hyperechogenic and cystic tumoral areas as hypoechogenic compared to adjacent parenchyma, enabling visibility of low and high grade gliomas.^110,111^ Woydt et al. identified solid tumor tissue in 89% (47 of 53) of biopsies taken from central areas of high grade and low grade gliomas, 72% (34 of 53) of which were inconspicuous on microscopic assessment. Furthermore, iUS enabled detection of histologically validated residual tumor in 22 of 25 cases in which gross total removal was suspected. Collectively, these findings suggested potential efficacy of iUS to delineate central areas of tumors compared to tumor rims, as well as to detect residual tumor that is indistinct by initial microscopic evaluation. However, iUS detection of hyperechoic rims was not specific to tumor tissue, as 44% (11 of 25) of biopsies were histologically assessed as normal brain tissue. Results from a more recent study demonstrate inferiority of iUS to detect tumor remnants after resection compared to iMRI.^112^ Additionally, interuser variability, low signal-to-noise ratio, and low resolution have limited further applications of iUS. To address these limitations, different approaches have been created to improve iUS. One of the more detailed approaches was performed by Liang et al. who overlayed contrast-enhanced iUS images with preoperative MRI images, which improved gross total resection rate from 31.58% (6 of 19 cases) to 84.62% (22 of 26 cases) and improved post-operative morbidity.^113^ As reviewed by Del Bene et al.,^114^ numerous other studies have attempted to use contrast-enhanced iUS, Doppler iUS, and multimodal imaging overlays with iUS. No randomized controlled trials have been completed to definitively determine whether any iUS method provides significant benefit for delineating gliomas, detecting residual tumor tissue, or improving overall survival or morbidity compared to other intraoperative imaging techniques, thereby limiting confidence for which iUS can be used clinically. iUS is therefore used as an adjunct imaging modality to supplement iMRI and other intraoperative methods and to repeatedly image resections after brain shift.

### Fluorescence-Guided Surgery

Fluorescence-guided surgery (FGS) is an intraoperative imaging modality that has become a mainstay of treatment world-wide for GBM, as it has been shown to significantly increase the EOR.^115–117^ The main advantage of FGS over conventional iMRI or image-guided technologies is the ability to provide immediate, intraoperative feedback regarding the surgical field of view while the neurosurgeon continues to actively perform surgery. As such, FGS does not encounter intraoperative accuracy problems due to brain shift, which is a major problem with image-guided technologies using pre-operative MRI, or the delayed, one-time feedback from iMRI.^56^

**Figure 5.**
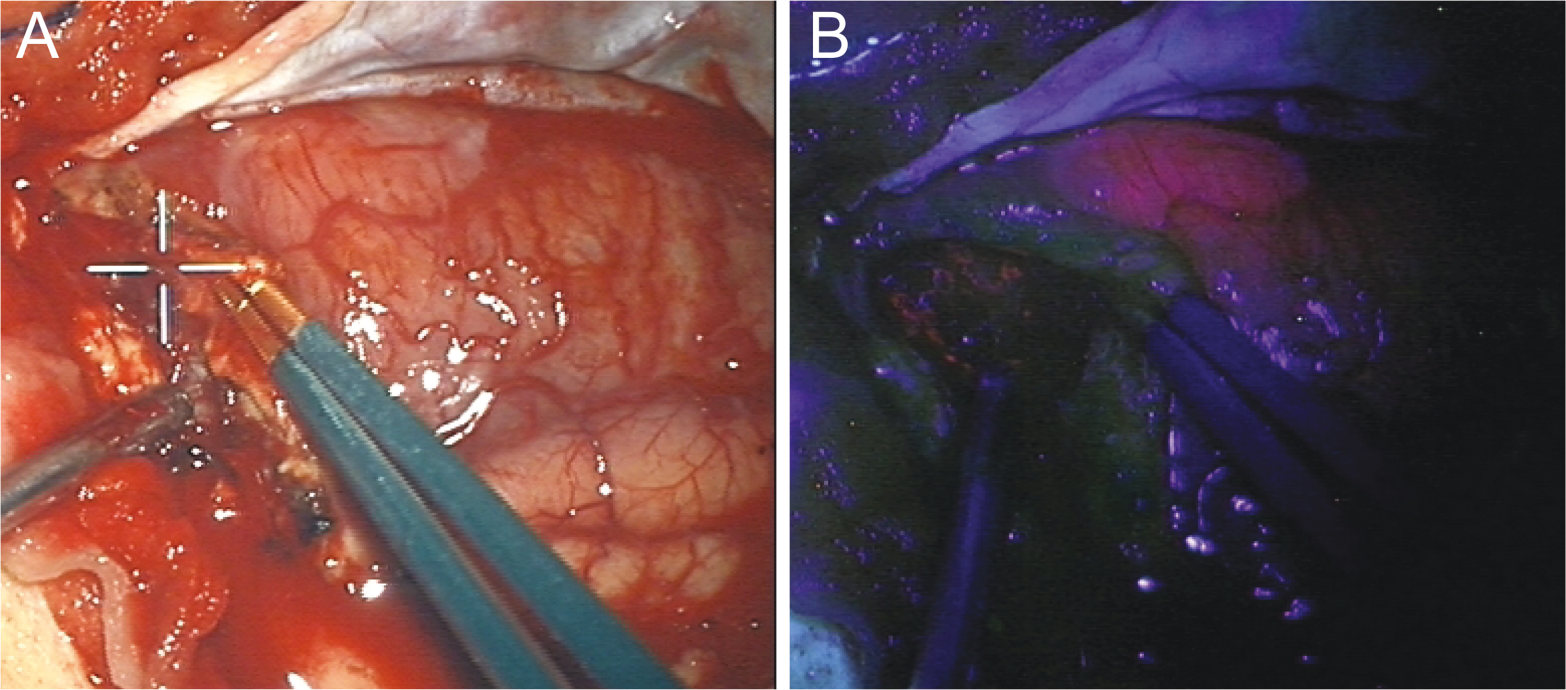
Fluorescence guided surgery using 5-ALA induced PpIX. Intraoperative images of a GBM patient undergoing 5-ALA induced PpIX fluorescence guided surgery. (A) Conventional white light image and (B) fluorescence image of the same surgical field of view as (A), demonstrating a region with red-pink fluorescence corresponding to a tumor tissue with significant accumulation of the tumor biomarker, PpIX.

In FGS, patients are administered a drug prior to surgery, which leads to significant accumulation of a fluorescent biomarker in tumor tissues.^116,118^ The three most used drugs are 5-aminolevulinic acid (5-ALA), indocyanine green (ICG), and fluorescein.^119,120^ 5-ALA leads to endogenous formation of protoporphyrin IX (PpIX), which is a fluorescent tumor biomarker that emits a red-pink fluorescence in the visible range of the spectrum of light (620–710 nm). Fluorescein emits a green-yellow fluorescence with a maximum fluorescence at 521 nm. ICG is a near-infrared agent, and as such is invisible to the human eye, emitting a maximum fluorescence at 835 nm.^121,122^ During surgery, neurosurgeons use a surgical microscope modified with light sources for exciting the fluorophores, appropriate filters to selectively collect the emitted fluorescent light, and sensors such as color cameras or near-infrared enabled cameras. These fluorescence-enabled microscopes allow the surgeon to visualize the fluorescence light emitted from tumor infiltrated tissue either with their eyes, using surgical oculars, or digitally by means of camera detection.^116,119,120^ Tumor infiltrated tissue will then display a distinct color that enables the surgeon to differentiate it from adjacent, normal brain. Figure 5 demonstrates an example of a patient undergoing 5-ALA FGS, with a conventional white light image on the left (Figure 5A), and a fluorescence image on the right (Figure 5B), showing the violet-blue background from tissue illumination and the red-pink 5-ALA-induced PpIX fluorescence emitted from a nodular tissue region.

A pivotal phase III clinical trial comparing standard of care resection using conventional white light guided resection versus FGS with 5-ALA, showed that FGS almost doubled the EOR (65% vs. 36%) and significantly increased the overall 6-month progression free survival of patients (41% vs. 21.1%).^117^ 5-ALA is the most widely used agent for FGS in GBM, with approval in the USA and Europe. However, the first FGS studies in neurosurgery date back to 1948 with the use of fluorescein, which accumulates in extracellular spaces and in areas of a broken blood brain barrier.^120,123^ ICG, which historically has served as a fluorescence agent for cerebrovascular imaging in neurosurgery, has seen an increase in research studies applied to GBM. Recent studies use what is known as second window ICG imaging, which entails administration of ICG 24 h prior to surgery. Then, likely due to biological processes dependent on the enhanced permeability and retention effect, ICG significantly accumulates in GBM, allowing for strong tumor-to-background signal and differentiation of tumor tissue from normal brain.^121^ In addition, several neurosurgical trials are evaluating novel FGS agents, adding to the already existing armamentarium of 5-ALA, ICG, and fluorescein, which includes endothelial growth factor receptor (EGFR) targeting antibodies, EGFR-targeting affibodies, protease activated fluorescent agents, and fluorophore labeled peptides.^115^

FGS has been shown to be a useful adjunct for maximizing the EOR in GBM surgery, though still has its limitations. First, current approaches using 5-ALA are limited to just surface assessments of tissue fluorescence. Near-infrared approaches such as ICG are also limited to depth assessments of tissue fluorescence of approximately 1 cm in depth.^121,122^ Second, each fluorescent biomarker may have a differing tumor-targeting profile that is dependent on the biology of the tumor tissue.^115,119,121,122^ Third, assessment of tissue fluorescence to date are all based on subjective, non-quantitative assessments of the raw, visible fluorescence observed by the naked eye or detected by a digital camera. These technologies do not take into account the heterogeneous effects of how light interacts with different tissues, which can lead to assessments of tissue fluorescence which are inaccurate (i.e., low sensitivity). These inaccurate assessments do not detect diagnostic levels of fluorescent tumor biomarker and may ultimately leave significant amounts of residual tumor tissue unresected.^116,119,124,125^ Finally, fluorescence agents to date provide the surgeon only with immediate structural feedback, but not with immediate functional feedback to differentiate functional from non-functional brain parenchyma (which can be provided only with direct electrical stimulation). As such, surgical resection based purely on FGS can lead to undesirable functional deficits if not properly used.^126^

To summarize, FGS has become a useful adjunct for improving the EOR in GBM. FGS provides the neurosurgeon with immediate feedback of the surgical field, distinguishing tumor infiltrated tissue from normal tissue, meanwhile the surgeon continues to actively perform surgery. However, like any surgical adjunct, the neurosurgeon needs to clearly understand its limitations to perform the most extensive tumor resection, meanwhile ensuring preservation of functional brain.

### Imaging After Surgery

**Figure 6.**
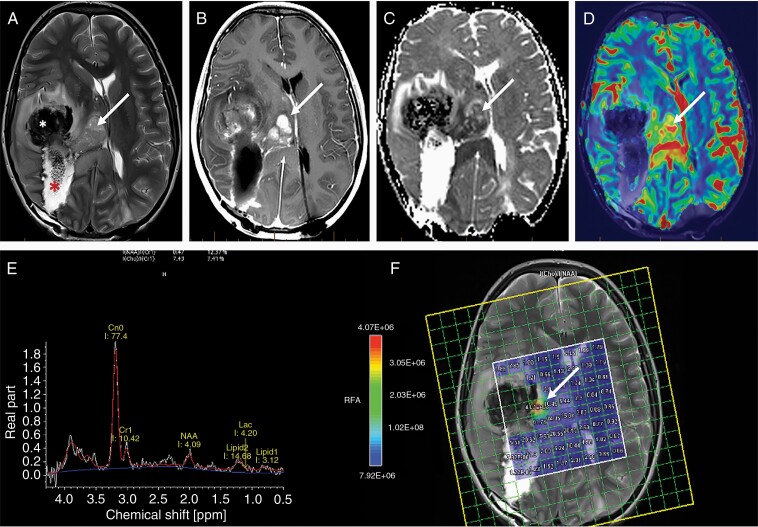
Imaging appearance of a glioblastoma following partial resection. Axial T2-weighted images (A) demonstrate a large hematoma (white asterisk) at the anterior aspect of the larger resection cavity (red asterisk) in the right parietal lobe. The arrow points to a residual T2 heterogenous component that demonstrates nodular enhancement (arrow) on the axial post contrast T1 weighted image (B) at the same level. The enhancing tissue demonstrates low ADC values (arrow) (C) suggesting hypercellularity; high cerebral blood volume (CBV) (D) (arrow) suggesting hypervascularity; tall choline peak compared to NAA (E) and a high choline/NAA ratio (F) on MR spectroscopic imaging.

In the postoperative setting, MR imaging is typically performed within 72 h to evaluate the extent of surgical resection and to identify potential surgical complications such as subacute hemorrhage and/or ischemia.^127^ It is important to diagnose infarct at the surgical margin at this early stage because subacute infarct can mimic progressive tumor on imaging. Immediate post-operative MRI is often used for planning of radiation therapy (Figure 6). This immediate post-operative imaging is also important in helping to differentiate between true tumor progression, pseudoprogression, or additional treatment effects when developing future therapeutic strategies. Various strategies (detailed below) have been proposed for interpretation of these imaging techniques. In addition, it is important to keep in mind that some therapies such as implantable BCNU wafers are known to change signal intensities and enhancement within the resection bed.^128^

### Imaging During Radiation Therapy

Even though the immediate post-operative MRI is routinely used for radiation planning, recent data suggest that this might not be the optimal practice. Some aggressive residual tumors can grow between the post-operative MRI and the start of radiation therapy. Additionally, the T2/FLAIR hyperintense areas around the resection margins, which is included in the radiation fields, often improves and normal brain tissue can be erroneously included in the field of view. Similarly, near the end of the radiation therapy schedule, the targeted tumor infiltrated tissue may shrink. This may not be readily appreciated, as intra-therapy imaging is not routinely performed. To circumvent this problem, many comprehensive cancer centers are routinely imaging the tumor to achieve near real-time adaptation of the radiation field with the role of imaging in radiation planning having been recently reviewed.^129^

## Imaging of Post-treatment GBM

### Assessment of Treatment Response

#### MacDonald Criteria.—

The MacDonald criteria, based on the largest cross-sectional diameter of contrast-enhancing tumors, has been used to assess tumor response via consecutively evaluating the lesions using MRI and includes steroid use and clinical findings which facilitate the classification of patients into four groups, namely, (a) complete response: disappearance of all enhancing lesions on consecutive MRIs at least one month apart, patient off steroids with stable or improved neurological exam; (b) partial response: >50% reduction in the size of the enhancing lesions on consecutive MRIs at least one month apart, patient on stable or reduced steroid dose with stable or improved neurological exam; (c) progressive disease: >25% increase in size of the enhancing lesions or appearance of new lesion, patient on stable or increased steroid dose with worsened neurological exam; or (d) stable disease encompassing all remaining radiographic and clinical scenarios.^130^ Responses according to these criteria need to be stable for >1 month. Notably, these criteria have been largely phased out in favor of the RANO criteria.

#### Response Assessment in Neuro-Oncology (RANO).—

The response assessment in neuro-oncology group has proposed standardized imaging practices for use in both clinical management and translational therapeutic protocols. In response to the failure of existing classification systems to account for (a) changes in enhancement secondary to corticosteroid use, antiangiogenic agents, postsurgical changes, radiation effects, and other inflammatory processes and (b) changes in the non-enhancing T2-hyperintense components secondary to use of antiangiogenic agents, Wen et al. introduced modified RANO guidelines for high-grade gliomas. These criteria measure enhancing lesions and additionally incorporate abnormalities in T2/FLAIR signaling to better differentiate between true tumor response and pseudoprogression or pseudoresponse in the setting of concomitant temozolomide and antiangiogenic therapy.^90^ Using these criteria, clinicians are more accurately able to distinguish between responses to therapies and progression of disease. By detailing timeframes with which to discuss treatment effects or failures, it allows time for the therapeutic agent, and in particular trial agents, to develop a more robust response before determining whether or not there is disease progression.^131^

#### Immunotherapy Response Assessment in Neuro-Oncology (iRANO).—

As described above, conventional therapies have done little to improve the survival of patients with GBM. As such, novel therapies are increasingly being investigated; immunotherapies are at the forefront of such approaches. Critically, patterns of imaging response to immunotherapies are quite unique from those of conventional therapies thereby necessitating the development of a response criteria capable of accounting for such differences. In line with this Okada et al. published the immunotherapy response assessment in neuro-oncology (iRANO) predominantly based on the experience of immunotherapies in non-CNS tumors.^132^ These criteria are better than RANO and have limited the premature withdrawal of subjects from clinical trials. However, given the complexity of immunotherapy centered approaches, it is remains difficult to use one set of criteria for all therapies/clinical trials. Results from recent clinical trails continue to shed light on how imaging can be used for assessment of novel immunotherapies.^133^

#### Response Assessment in Pediatric Neuro-Oncology (RAPNO).—

Since the inception of the RANO criteria, it has been used almost exclusively for assessment of GBM, including pediatric GBM. However, it is prudent to note that the biology of high-grade gliomas in children is different from that in adults, and children frequently require different management. To mitigate these discrepancies, the Response Assessment in Pediatric Neuro-Oncology working group has recently published recommendations for response assessment for pediatric patients with high-grade gliomas.^134^

### Prediction of Response

Prediction of treatment response in GBM is immensely challenging. The enhancing components of GBM at presentation are inversely associated with survival^135^; similarly, residual contrast-enhancing tumor following surgery is also negatively associated with survival in patients with newly diagnosed GBM.^136^ Pretreatment ADC histogram and CBV-based thresholding values have been shown to predict response in recurrent GBM treated with antiangiogenic therapies.^137,138^ Early reduction of CBV following bevacizumab treatment in recurrent glioblastomas can predict survival as well.^139^

### Assessment of Pseudoprogression vs. True Tumor Progression

Pseudoprogression is a well-documented phenomenon observed in 20–30% of patients in which new tissue enhancement is detected within three months of completion of radiation with concurrent temozolomide therapy in the absence of true tumor growth, as determined by the presence of necrosis or gliosis on biopsy.^140,141^ The mechanism of pseudoprogression remains poorly understood, but it is thought to represent edema and increased vascular permeability secondary to chemoradiotherapy-induced tumor and endothelial cell death. Notably, O6-methyl guanine-DNA methyl transferase (MGMT) methylated tumors have a higher incidence of pseudoprogression due to their increased sensitivity to temozolomide.^90,130,141–145^ Pseudoprogression is also expected with targeted therapy and/or immunotherapy.^146^ Pseudoprogression carries significant clinical impact as studies have shown this subgroup of patients experience improved median survival if correctly identified.^145,147,148^ Pseudoprogression typically stabilizes or resolves without a corresponding change in treatment. The apparent enlargement of the tumor on MRI due to pseudoprogression can be difficult to reliably distinguish from true tumor progression although patients with pseudoprogression are less likely to experience worsening of neurological symptoms. Critically, the ability to distinguish pseudoprogression has become ever more relevant in the era of immunotherapy.

MRI is essential in assessing response to treatment and tumor progression during the disease course. The ability to identify surrogate imaging biomarkers allows for a noninvasive way to risk-stratify patients and to refine individual therapeutic approaches. An overview of the techniques currently in use along with their specific advantages and limitations is provided below.

#### Delta T1 Map.—

**Figure 7.**
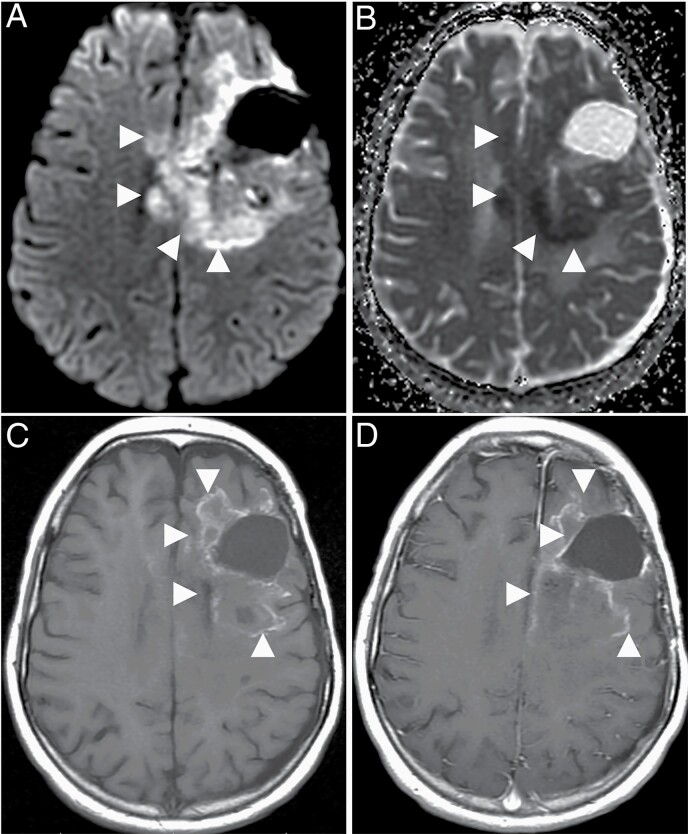
Imaging appearances of a glioblastoma following treatment with bevacizumab. Axial diffusion weighted image (A) through the level of centrum semiovale demonstrates a confluent area of high signal (arrowheads) surrounding the cystic resection cavity associated with low values (arrowheads) on the corresponding ADC map suggestive of diffusion restriction developed after treatment with bevacizumab. The precontrast axial T1-weighted image (C) at the same level demonstrates irregular T1 hyperintensities. Enhancement, if any, is hard to appreciate on the post contrast T1 weighted image (D) at the same level, demonstrating the importance of the delta T1 map (not shown).

Delta T1 map can be very helpful in evaluation of enhancement in the post treatment setting, particularly after treatment with bevacizumab that induces intratumoral heterogenous T1 hyperintensity^149,150^ (Figure 7). Moreover, the delta T1 map can also predict survival with bevacizumab therapy.^149^

#### DWI.—

ADC can aid in differentiating radiation-induced effects from tumor progression or recurrence.^90,130,143,144^ Enlarging tumors usually have lower ADC (~1.0–1.3 µm^2^/ms) compared with pseudoprogression (>1.3 µm^2^/ms).^151,152^ In the presence of extremely heterogeneous tumor microenvironments, the presence of therapy-related hemorrhage confounds the ADC measurement. Similarly, bevacizumab therapy is associated with a distinct persistent diffusion restriction (Figure 7) that is associated with better survival and can mimic diffusion restriction related to tumor recurrence.^153^ DWI alone therefore inadequately differentiates pseudoprogression from GBM progression.

#### Perfusion MRI.—

Perfusion MRI has also been successfully used to differentiate pseudoprogression from true tumor progression with estimated sensitivity and specificity approaching 90%.^154^ Median CBV is usually lower in pseudoprogression compared to true tumor progression.^152^ Fractional tumor burden (FTB) calculation is a technique of histogram-based thresholding that can be an excellent tool to quantitatively differentiate pseudoprogression from true tumor progression and predict survival.^155,156^ A simple dichotomous “true progression” and “pseudoprogression” approach is challenging in clinical practice because enlarging enhancement, most of the time, is contributed to by both tumor tissue and tissue with treatment related effects. Unfortunately, if a voxel contains both tumor tissue and tissues with pseudoprogression, the sensitivity and specificity of perfusion imaging in differentiating pseudoprogression from true tumor progression decline. Additionally, there is wide variability in DSC-MRI acquisition, and post processing approaches can contribute to variation in results. Standardized acquisition techniques and post processing techniques have been recently recommended for consistency and inter-institutional comparability.^157,158^

#### Density-based Magnetic Resonance Image Clustering for Assessing Tumor Heterogeneity (DEMARCATE).—

DEMARCATE is a novel MRI-based technology used to analyze tumor heterogeneity.^159^ This method generates a tumor density profile comprising voxel intensities corresponding to specific regions within the tumor. Probability density functions applied in a Fisher–Rao Riemannian framework are used for metric-based clustering of patients. The outputted patient clusters demonstrate significant associations with tumor morphology, driver gene mutations, and prognostic clinical outcomes and additionally map with known GBM subtypes (cluster 1 with proneural and cluster 2 with mesenchymal, neural, and classical).^160^ Whereas most methods employ scalar summary measures to analyze tumor heterogeneity, DEMARCATE gains predictive and correlative power by using the entire density of an individual tumor density profile to capture highly refined information that can be used to detect small-scale and sensitive changes in the tumor due to treatment effects.

#### MR Spectroscopy.—

MR spectroscopy has been used to identify tumor progression as well. Chuang et al. have also shown that Cho/Cr and Cho/NAA ratios are significantly higher in tumor recurrence compared with radiation injury.^161^

#### Quantitative MRI.—

Itakura et al. identified three distinct phenotypic GBM subtypes utilizing a quantitative MR imaging analysis of lesion shape, texture, and edge sharpness with subsequent consensus clustering. The three clusters, pre-multifocal, spherical, and rim-enhancing, mapped to distinct sets of molecular signaling pathways (e.g., c-Kit, FOXA) using molecular activity estimates from The Cancer Genome Atlas and notably demonstrated differential probabilities of survival.^162^

#### PET Imaging.—

PET imaging is increasingly used in situations when MRI inadequately differentiates pseudoprogression from true progression. Although 18FDG PET has been used in this setting, LNAA PET can reliably distinguish tumor progression from treatment-related effects and can in turn identify responders to antiangiogenic therapy with near complete diagnostic accuracy.^163–165^ The European Association of Nuclear Medicine (EANM), the Society of Nuclear Medicine and Molecular Imaging (SNMMI), the European Association of Neurooncology (EANO), and the working group for Response Assessment in Neurooncology with PET (PET-RANO) have jointly published guidelines for appropriate use of PET imaging in evaluation of gliomas.^166^

### Assessment of Pseudoresponse

**Figure 8.**
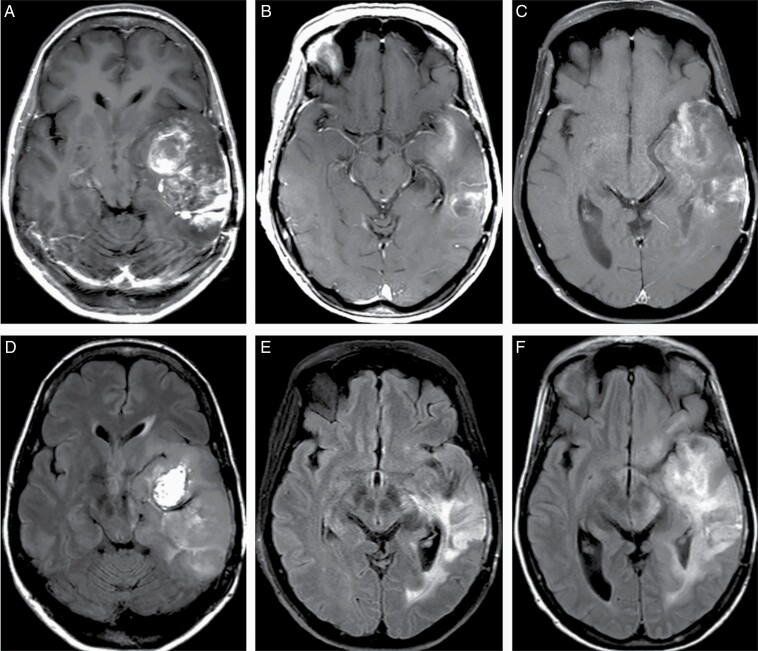
Pseudoresponse following bevacizumab therapy in a 15 year old male patient with recurrent high grade glioma with histone H3.3 G34 mutation in the left temporal lobe. Extensive heterogenous enhancement (A, post-contrast T1-weighted image) and edema with mass effects (D, FLAIR image) have been significantly reduced 3 weeks after the start of bevacizumab therapy (B, E, post-contrast T1-weighted image and FLAIR image, respectively); however, there is evidence of worsening of enhancement (C, motion degraded post-contrast T1-weighted image) and infiltrative tumor component (F, FLAIR image), on a follow-up MRI obtained 8 weeks after the start of bevacizumab therapy.

Pseudoresponse describes the rapid reduction in tumor enhancement and surrounding vasogenic edema following the administration of an anti-angiogenic agent that is not representative of a true anti-tumor response. Treatment with bevacizumab, an anti-VEGF monoclonal antibody approved for use in GBM refractory to first line radiation, temozolomide, and lomustine, commonly causes pseudoresponse as it decreases microvascular proliferation and BBB permeability (Figure 8). Though the radiographic findings do not correspond to true tumor response, and treatment with bevacizumab does not confer any survival benefit, it has been associated with symptomatic improvement and reduced steroid dependence.^167^ This is likely the result of reduced mass effect and vasogenic edema. Of note, patients requiring a drug holiday due to toxicities have been shown to demonstrate rebound enhancement and edema with a subsequent “re-response” after restart.^168,169^

The differentiation between clinically distinct radiographic phenomena from a true tumor response has critical implications for patient care as failure to correctly identify tumor progression or regression may result in inappropriate modification or cessation of effective treatment.^140,141,170,171^

### Assessment of Radiation Necrosis

Radiation necrosis is a delayed manifestation of tissue injury that typically occurs three months to one year following radiotherapy. Radiation necrosis occurs as a result of disruption of the BBB with consequent occlusive vasculopathy, thrombosis, and ischemia. Histologic examination shows extensive glial and white matter damage, fibrinoid necrosis of small vessels, and vessel wall hyalinization.^172,173^ Radiographically, radiation necrosis presents as a heterogeneously enhancing lesion with a characteristic “soap-bubble” or “Swiss cheese” appearance and surrounding vasogenic edema. MR findings are useful in differentiation from both pseudoprogression and tumor progression. For example, development of a lesion in an area of radiation-induced leukoencephalopathy or in the bed of a previously non-enhancing tumor with minimal to no mass effect and associated hemosiderin and calcifications is suggestive of radionecrosis. In addition, an elevated lipid-lactate peak on spectroscopy and a low normalized cerebral blood volume (CBV) ratio, a biomarker for angiogenesis, also carry diagnostic value.

## Emerging Imaging Techniques

### Mass Spectrometry Imaging

Technological ingenuity over the past several decades has rendered *ex vivo* mass spectrometry imaging (MSI) a feasible and valuable tool in guiding intraoperative decision-making during tumor resection.^174,175^ MSI is an imaging modality that analyzes the molecular composition of thin tissue sections based on the mass-to-charge ratios of the ionized compounds that constitute the surface. Unlike traditional liquid chromatography methods, MSI does not require chromatographic purification and thus preserves spatial information and tissue architecture. The two most common ionization techniques for surface analysis of biological tissue are matrix-assisted laser desorption ionization (MALDI) and desorption electrospray ionization (DESI).

MALDI and DESI-MSI have numerous applications in GBM research. MALDI-MSI has been used to detect in situ, spatial histone variation in patient-derived xenograft models,^176^ which may provide novel genomic targets for antibody-mediated therapy. DESI-MSI has also been used to analyze the ganglioside composition of healthy and malignant nervous tissue and could reliably distinguish between tumor, grey matter, and white matter, while also identifying ganglioside forms present exclusively in GBM tissue.^177^ Looking forward, a multicenter effort to investigate GBM tissue protein composition using liquid chromatography-tandem mass spectrometry could be used to construct a GBM proteome and identify actionable serum GBM biomarkers.^178,179^ MSI has additional functionality in determining tissue penetration and efficacy of various therapeutic agents, e.g. boron neutron capture therapy relies on fission reactions to selectively kill tumor cells that have reached a sufficient intracellular density of boron, a measure that can be reliably studied using secondary ion MSI.^180^ In addition, the efficacy of oncolytic viral therapy in GBM treatment is highly dependent upon the selective targeting of tumorigenic cells, which can be assessed with MALDI-MSI.^181^

### Artificial Intelligence

In recent years, there has been great interest in applying artificial intelligence (AI) within multiple medical fields, including imaging. Sotoudeh et al. have recently reviewed AI techniques in the context of brain tumor imaging.^182^ In short, supervised and unsupervised machine learning and different neural network techniques can be used to train algorithms via a series of known values (e.g., imaging, histological, genetic, and clinical substrates in variable combinations) thereby facilitating the prediction outcomes based on initial training datasets.

Interestingly, AI has been employed as a method to automate complex imaging analyses in an unbiased and reproducible manner. Machine learning is a subfield of AI where machines can perform pattern recognition without any explicit instruction. In this way, patterns and models may be generated based on datasets without the influence or unconscious bias of human input. Given sufficiently large datasets, machines are able to determine the optimal combination of relevant features to explain a given phenomenon. For example, machine learning can determine radiomic parameters that may be present in histopathologically and molecularly distinct subgroups of glioma in any given imaging modality. This abstraction becomes especially powerful given our relative inability to process a near infinite volume of voxel data.^183^ Importantly, these methods are able to be employed in even the most complex of tumoral environments. Convolutional neural networks are among the most powerful machine learning techniques applied to imaging interpretation in GBM. They are able to operate without any human training. By eliminating human error, deep convolutional neural networks have been used to differentiate glioblastoma subcompartments based on MR imaging by generating rapid and accurate three-dimensional segmentations of the tumor. Radiomic features have been identified that accurately predict survival in patients with GBM.^184^ Radiomic features derived from these segmentations are particularly useful in helping to predict genetic biomarkers.^185,186^ For example, artificial intelligence has been used to predict IDH, ATRX, and CDKN2 family mutations in addition to chromosome 7/10 aneuploidies with astonishing sensitivity and specificity.^187^ Another promising application of artificial intelligence was demonstrated with stimulated Raman imaging, a form of optical imaging, where clinicians showed that a convolutional neural network driven stimulated Raman imaging technology had a noninferior performance compared to pathologist interpretation of histologic images for tissue diagnosis during brain tumor surgery (94.6 % vs. 93.9% accuracy).^188^ While still in its infancy, AI represents a promising future direction for imaging analysis in patients with GBM and may ultimately prove helpful in multiple aspects of brain tumor management, including tumor diagnosis/grading, the prediction of genomic and histopathologic architecture, and/or operative/radiation planning.

## Conclusion(s)

In the last two decades, there have been significant changes in the way GBM is imaged. Advanced imaging techniques now play a significant role in the current diagnostic and treatment paradigms. Diffusion and perfusion MRI are now routinely used for diagnosis of brain tumors. DTI and fMRI in combination are now part of current standard(s) of care for brain mapping prior to surgical resection. Intra-operative MRI, ultrasound, and fluorescent techniques are increasingly being adopted for more precise tumor surgery with significantly better operative outcomes. Advanced MRI techniques are also routinely used for assessment of treatment responses which is critical in the era of biologic/immune centered therapies. Such advanced imaging modalities are critical and physicians, surgeons and scientists look to advance treatments and improve clinical outcomes for patients in dire need.
